# Population heterogeneity in *Mycobacterium smegmatis* and *Mycobacterium abscessus*

**DOI:** 10.1099/mic.0.001402

**Published:** 2023-10-20

**Authors:** Sarah E. M. Born, Matthew J. Reichlen, Iona L. Bartek, Jeanne B. Benoit, Daniel N. Frank, Martin I. Voskuil

**Affiliations:** ^1^​ Department of Immunology and Microbiology, University of Colorado Anschutz Medical Campus, Aurora, Colorado, USA; ^2^​ Division of Infectious Diseases, Department of Medicine, University of Colorado Anschutz Medical Campus, Aurora, Colorado, USA

**Keywords:** *Mycobacterium smegmatis*, *Mycobacterium abscessus*, heterogeneity, Lsr2, morphology

## Abstract

Bacteria use population heterogeneity, the presence of more than one phenotypic variant in a clonal population, to endure diverse environmental challenges – a ‘bet-hedging’ strategy. Phenotypic variants have been described in many bacteria, but the phenomenon is not well-understood in mycobacteria, including the environmental factors that influence heterogeneity. Here, we describe three reproducible morphological variants in *M. smegmatis* – smooth, rough, and an intermediate morphotype that predominated under typical laboratory conditions. *M. abscessus* has two recognized morphotypes, smooth and rough. Interestingly, *M. tuberculosis* exists in only a rough form. The shift from smooth to rough in both *M. smegmatis* and *M. abscessus* was observed over time in extended static culture, however the frequency of the rough morphotype was high in pellicle preparations compared to planktonic culture, suggesting a role for an aggregated microenvironment in the shift to the rough form. Differences in growth rate, biofilm formation, cell wall composition, and drug tolerance were noted among *M. smegmatis* and *M. abscessus* variants. Deletion of the global regulator *lsr2* shifted the *M. smegmatis* intermediate morphotype to a smooth form but did not fully phenocopy the naturally generated smooth morphotype, indicating Lsr2 is likely downstream of the initiating regulatory cascade that controls these morphotypes. Rough forms typically correlate with higher invasiveness and worse outcomes during infection and our findings indicate the shift to this rough form is promoted by aggregation. Our findings suggest that mycobacterial population heterogeneity, reflected in colony morphotypes, is a reproducible, programmed phenomenon that plays a role in adaptation to unique environments and this heterogeneity may influence infection progression and response to treatment.

## Introduction

Population heterogeneity is a common and well-documented phenomenon within both saprophytic and pathogenic bacteria [1]. Variation leads to selective advantage for minority subpopulations in rapidly changing environments and can contribute to a population better equipped to handle diverse challenges compared to a homogenous population [2]. Non-mutational genetic changes, epigenetic alterations, and protein inheritance, as well as spontaneous mutation contribute to population heterogeneity. Variants generated by non-mutational genetic and epigenetic mechanisms are typically reversible, although downstream signalling events can render the variant switching irreversible [3]. Population heterogeneity contributes to competence and sporulation in *Bacillus subtilis* [4, 5], antigen variation in *Neisseria gonorrhoeae* [6] and *Salmonella* Typhimurium [7], and changes in virulence and colonization dynamics in *Streptococcus* species [8, 9]. We previously described non-mutational heterogeneity in *Burkholderia pseudomallei *, in which three colony morphotypes displayed divergent virulence and ability to colonize the gastric tract [10].

Spontaneous shifting from smooth to rough variants has been noted in multiple bacterial species including mycobacteria [11–13] and is known to affect virulence and infectivity. Nontuberculous mycobacteria primarily exist in a smooth form in the environment, while rough forms have been documented in *M. smegmatis* [14], *M. abscessus* [15], and *M. avium* [16], primarily coming from infection models or patient samples [17]. *M. tuberculosis* has been described only as having a rough form [18]. Rough colony variants in mycobacteria typically lack glycopeptidolipids [16, 19] which are cell-surface-associated lipids found on nontuberculous mycobacteria. Glycopeptidolipids are known to be involved in sliding motility, bacterial aggregation, biofilm formation, phage susceptibility, and cellular morphology, as well as survival in host cells. The lack of glycopeptidolipids also alters how the immune system interacts with the bacteria, allowing for innate immune signalling through TLR2 and a more inflammatory response [20]. Rough variants in non-tuberculous mycobacteria are hyper-aggregative and lack sliding motility [19, 21] and have been noted to be more susceptible to mycobacteriophage, with no smooth strains reported to be killed by phage [22].

Rough forms in *M. abscessus* and *M. avium* generally exhibit a more invasive phenotype compared to smooth forms, and display characteristics associated with higher virulence such as cording and hyper-aggregation [23]. They have increased infectivity in macrophage and mouse models [24], similar to observations in other bacteria. The presence of rough forms correlates with more severe disease symptoms and rough isolates occur with greater frequency as chronic lung infection progresses over time [25, 26]. Interestingly, a rough strain of *M. smegmatis* was also able to persist longer in a macrophage model compared to the smooth parent form [27]. *M. canettii*, a member of the *M. tuberculosis* complex that exists primarily as a smooth form, is rarely found in the context of infection and cases of *M. canettii* tuberculosis generally come from environmental sources as opposed to human-to-human transmission [18]. Of note, a spontaneous rough mutant of *M. canettii* was reported to have higher virulence than the smooth parental isolate [28].

Rough and smooth variants described in mycobacterial species are often attributed to mutation, with rough strains carrying mutations in *mps1*, *mps2* [27], *mmpL4a*, *mmpS4* [29], *rel,* or *dcpA* [30] and smooth variants with mutations in *aceE* [31], *rnj* [32], or *lsr2* [33]. While many colony variants in mycobacteria do have mutations, in this paper we propose that mutation is not necessarily the sole mechanism by which colony variants form, but, like the yellow variant of *B. pseudomallei* that forms under hypoxic stress, epigenetic changes can also occur to push the bacterium into a smooth phenotypic variant. Indeed, while many of these mutations do produce rough or smooth colonies, the resulting strains do not encompass all the phenotypic characteristics of the spontaneous rough or smooth variants.

In this paper, we characterize three semi-stable phenotypic variants in *M. smegmatis *, which range from smooth and hydrophilic (A morphotype), to rough and hydrophobic (C morphotype), with an intermediate morphotype (B morphotype) predominating under standard laboratory planktonic growth conditions. These morphotypes are unlikely to be attributable to random mutation, which was supported by whole genome sequencing analysis. The three forms have distinct growth rates and glycopeptidolipid profiles and while the forms respond similarly to some drugs, there are distinct drug-response phenotypes. We also demonstrate that the *M. abscessus* rough form has striking phenotypic similarities to the *M. smegmatis* C form and that both rough forms can be reproducibly generated through pellicle formation. While this paper does not address all forms of population heterogeneity within mycobacterial species, the phenomenon of repeatable form generation through culture and pellicle formation is one facet involved in overall population heterogeneity. Other mechanisms of colony heterogeneity include mutations accumulated during other environmental conditions including infection but were not observed in our standard *in vitro* conditions.

## Methods

### Culture conditions and strains

For gross microscopy and morphology characterization, *M. smegmatis* mc^2^155 forms A, B, and C and *M. abscessus* ATCC19977 rough and smooth forms were grown in Dubos medium (BD Difco) supplemented with 0.5 % bovine serum albumin (Research Products International), 0.05 % Tween 80, 0.75 % glucose, and 0.17 % sodium chloride, pH 6.6 (DTA medium) in 125 ml Erlenmeyer flasks overnight, then diluted in DTA medium and plated to Luria-Bertani Lennox (LB) agar (Fisher BioReagents). Light microscopy was performed using an Omax compound microscope with ToupView 3.7 software after 2 days of incubation at 37 °C. Pellicles were generated by growing the bacteria stirring overnight in DTA, diluting in Dubos broth made without detergent, and settling at 37 °C until a robust pellicle formed.

To determine growth rate, A, B, and C forms of *M. smegmatis* mc^2^155 were grown at 37 °C overnight in DTA medium in 125 ml Erlenmeyer flasks. Cultures were then diluted to an optical density at 600 nm (OD_600_) of 0.05 in 50 ml DTA medium and started stirring at 150 r.p.m. OD_600_ was measured throughout the growth curve for 24 h.

For drug challenge, cultures of A, B, and C forms of *M. smegmatis* mc^2^155 were grown in DTA medium overnight. Cultures were then diluted to an OD_600_ of 0.05 and added to 14 ml snap-cap tubes in 1 ml aliquots. These tubes were incubated shaking at 250 r.p.m. at 37 °C for 1 h before drugs were added. Tubes were returned to shaking at 250 r.p.m. at 37 °C. After 6, 24, or 48 h depending on experiment, each sample was serially diluted in DTA medium and plated to DTA agar supplemented with OADC and 0.4 % activated charcoal to determine survival. For *M. abscessus* ATCC19977 drug challenge, rough and smooth forms were grown in DTA medium overnight. These cultures were diluted to an OD_600_ of 0.02 and added to 14 ml snap-cap tubes in 4 ml aliquots. These tubes were incubated shaking at 250 r.p.m. at 37 °C for 4 h before drugs were added. Tubes were returned to shaking at 250 r.p.m. at 37 °C. After 24 and 48 h, each sample was serially diluted and plated to DTA agar supplemented with OADC and 0.4 % activated charcoal to determine survival.

### Genomic analysis

Genomic DNA was extracted from each *M. smegmatis* variant (see Table 1 for isolate details). A genomic library was generated for each variant using the Illumina Nextera XT kit to fragment DNA and add adapters. Genomic libraries were sequenced on the Illumina MiSeq platform using the MiSeq Reagent Kit v3 (2×300 cycle). Isolate genomes were analysed for sequence variation using *breseq*, which is a computational pipeline that uses reference-based alignment approaches of short-read sequencing data to identify mutations in a sample compared to the refence genome [34], which was *M. smegmatis* mc^2^155 for this project. Potential causative mutations were called when a mutation existed in all isolates of the same form, but in none of the isolates of the other forms. Raw sequencing data can be accessed through the BioProject Accession PRJNA802871.

**Table 1.** *
M. smegmatis* morphotype isolates for sequence analysis

Isolate	Morphotype lineage	no. of reads
SB2016A1	A pure culture	763 982
SB2016B2	B pure culture	1 010 217
SB2016C3	C pure culture	993 428
SB2016AB4	A switched to B	1 078 770
SB2016AB5	A switched to B	493 406
SB2016BA6	B switched to A	272 036
SB2016BA10	B switched to A	921 363
SB2016BC8	B switched to C	715 799
SB2016BC9	B switched to C	1 218 765

### Construction of *lsr2* deletion mutant

The *lsr2* deletion strain in *M. smegmatis* mc^2^155 was constructed as previously described for *M. tuberculosis* [35, 36]. Briefly, flanking regions upstream and downstream of *M. smegmatis lsr2* were amplified by PCR using the following primers: upstream forward primer 5′-TTTTTTTTCCATAGATTGGGCATTCCACGCGGCAACT-3′, upstream reverse primer 5′-TTTTTTTTCCATCTTTTGGCTTCCTTGCCCTGGTAGCC-3′, downstream forward primer 5′-TTTTTTTTCCATAAATTGGGCAATGAGGGTGTGGATTCC-3′, and downstream reverse primer 5′-TTTTTTTTCCATTTCTTGGCACTTTCTTTGCCATTCCCG-3′. Flanking regions of *lsr2* were cloned into the pAES0004S plasmid containing a hygromycin resistance cassette. The vector was then ligated into the phasmid phAE159. *M. smegmatis* mc^2^155 was electroporated with the phasmid and resulting phage was amplified to obtain high-titre stock. *M. smegmatis* mc^2^155 B form was infected with the high-titre phage and plated on DTA agar containing hygromycin. Hygromycin resistant colonies were picked and *lsr2* deletion was confirmed by PCR.

### Sliding motility assay

Sliding motility was assessed as previously described [37]. Briefly, semi-solid 7H9 agar (0.3%) with no additional carbon source was inoculated with a colony of each *M. smegmatis* mc^2^155 or *M. abscessus* ATCC19977 morphotype, sealed with parafilm, and incubated at 37 °C for 2 weeks.

### Biofilm assay

Biofilm formation was assessed as previously described [38, 39]. Briefly, *M. smegmatis* mc^2^155 and *M. abscessus* ATCC19977 forms were grown to mid-log phase in DTA medium, then diluted to OD_600_ 0.05 in Sauton minimal medium and incubated 48 h at 37 °C. Biofilms were stained with Gram safranin and quantified by absorbance at 550 nm after elution of the safranin into 30 % acetic acid and a 1 : 10 dilution.

### Glycopeptidolipid analysis

Lipids were analysed as described previously [40, 41]. Briefly, cultures were grown to mid-logarithmic growth phase in DTA and collected by centrifugation. Lipids were extracted from cell pellets using chloroform-methanol (2 : 1, v/v). Extracts were deacylated by alkaline methanolysis and glycopeptidolipids were resolved by thin-layer chromatography with a chloroform:methanol:water (90 : 10 : 1, v/v) solvent system. Glycopeptidolipids were visualized by spraying plates with 10 % sulphuric acid in ethanol, followed by charring.

### Statistical methods

Rates of growth and biofilm formation in *M. smegmatis *, as well as time-course drug experiments in both *M. smegmatis* and *M. abscessus* were evaluated by a one-way analysis of variance followed by a multiple comparison analysis of variance by a one-way Tukey test. Biofilm formation in *M. abscessus* was evaluated using a two-tailed, unpaired Student’s t-test. Differences were considered significant at the 95 % level of confidence (*P*<0.05). All statistical tests were performed using Prism 10.

## Results

### *M. smegmatis* and *M. abscessus* reproducibly formed distinct colony morphotypes

**Fig. 1.**
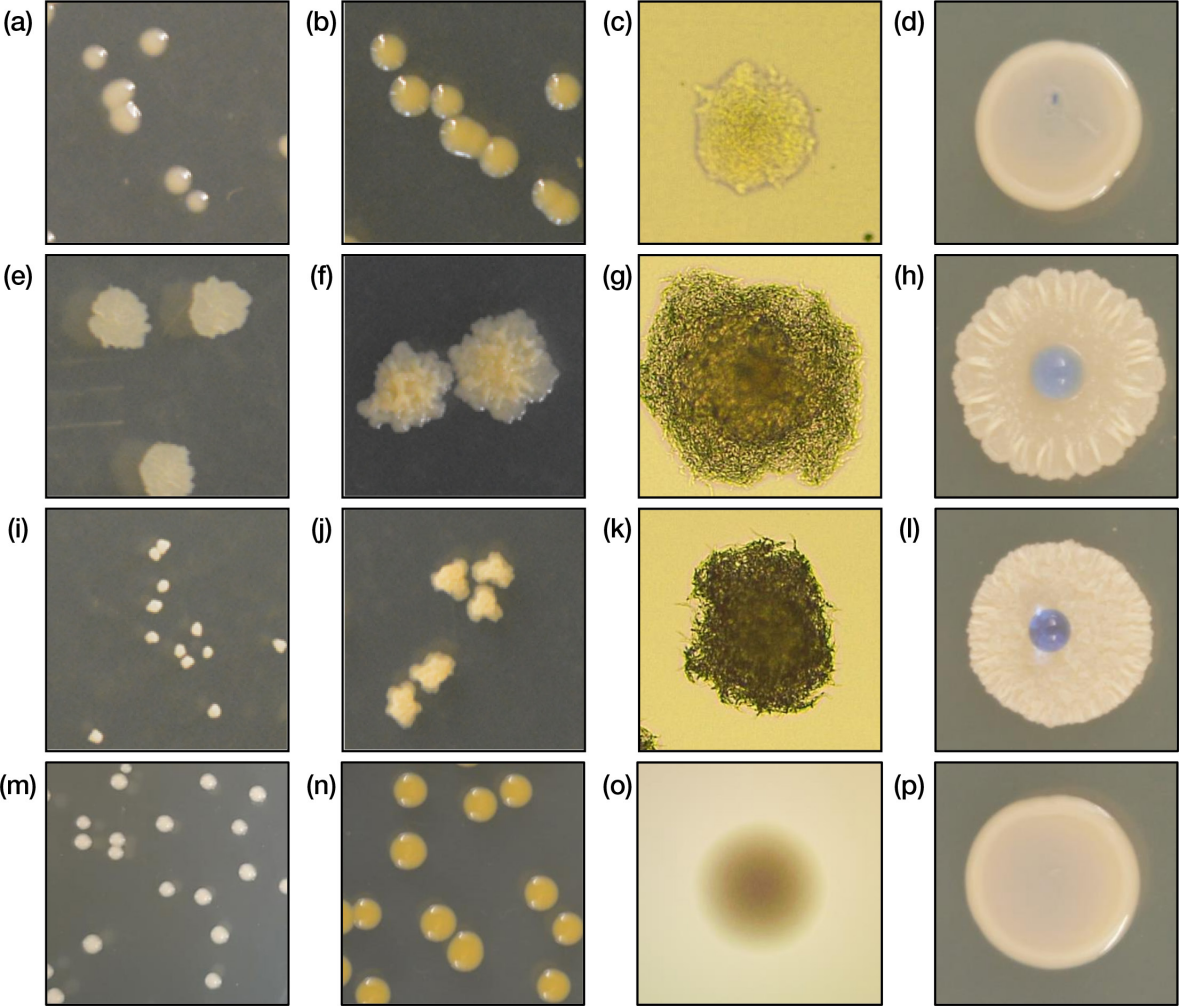
*
Mycobacterium smegmatis* exists in three semi-stable phenotypic variants. Morphotype A colonies were round, smooth, and hydrophilic. Morphotype B colonies were irregular, wrinkly, and moderately hydrophilic. Morphotype C colonies were irregular, hydrophobic, and raised. (**a-c**) Morphotype A after 3 days incubation at 37 °C, after seven additional days incubation at 23 °C, and under light microscopy after 2 days incubation at 37 °C using 10× objective. (**d**) Confluent morphotype A with 5 µl water in centre. (**e-g**) Morphotype B after 3 days incubation at 37 °C, after seven additional days incubation at 23 °C, and using light microscopy after 2 days incubation at 37 °C using 10× objective. (**h**) Confluent morphotype B with 5 µl water in centre. (**i-k**) Morphotype C after 3 days incubation at 37 °C, after seven additional days incubation at 23 °C, and using light microscopy after 2 days incubation at 37 °C using 10× objective. (**l**) Confluent morphotype C with 5 µl water in centre. (**m-o**) *M. smegmatis* Δ*lsr2* colonies after 3 days incubation at 37 °C, after seven additional days incubation at 23 °C, and using light microscopy after 2 days incubation at 37 °C using 10× objective. (**p**) Confluent *M. smegmatis* Δ*lsr2* with 5 µl water in centre.

We identified and examined the formation of three semi-stable colony variants from *M. smegmatis* cultures, referred to as A, B, and C in this paper. These variants were first isolated from 4 month-old settled cultures and all were also observed in logarithmically growing cultures at low frequencies. Using pellicle preparations, the C morphotype could be reproducibly enriched compared to logarithmically grown cultures. Morphotype A was small, round, and wet, and appeared translucent when viewed using light microscopy (Fig. 1a–c). Morphotype B was moist and flat with ridges in the colony. Using light microscopy, B was not as translucent as A and had a raised, out of focus centre. Ridges were apparent in the centre of B colonies, as could be seen by eye (Fig. 1e–g). Morphotype C was small, dry, and raised. Using light microscopy, it appeared black due to the density of the colony (Fig. 1i–k). The morphotypes also exhibited varying degrees of hydrophobicity. Morphotype A colonies were hydrophilic, C colonies were hydrophobic, and B colonies appeared hydrophilic but not to the degree of the A colonies (Fig. 1d, d, l). The three *M. smegmatis* forms were observed on LB, 7H10, and DTA solid media (Figs 1 and S1, available in the online version of this article).

**Fig. 2.**
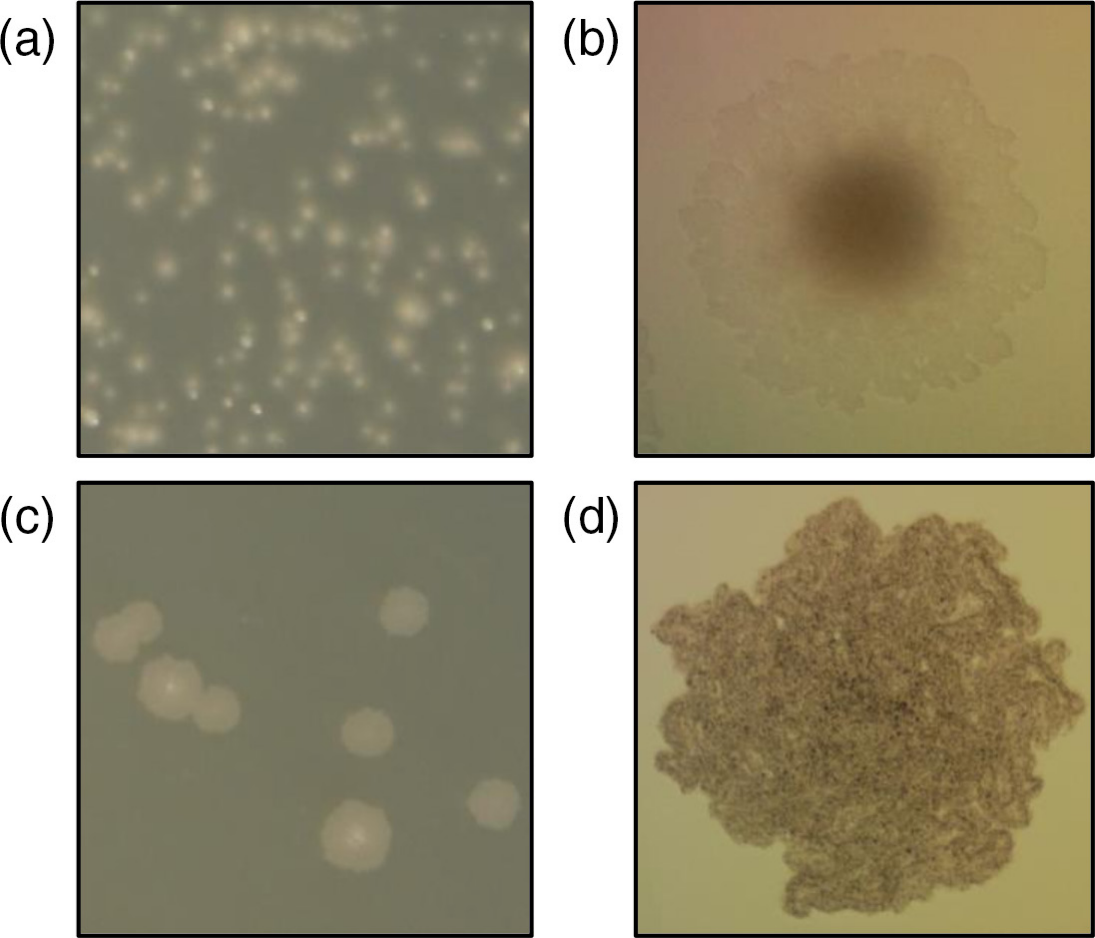
*
Mycobacterium abscessus* exists in two semi-stable phenotypic variants. Smooth colonies were round, domed, and wet. Rough colonies were irregular, wrinkly, and dry. Rough colonies appeared to exhibit a cording-like phenomenon when examined using light microscopy. (**a**) Smooth morphotype after 3 days incubation on LB agar at 37 °C. (**b**) Smooth morphotype under light microscopy after 2 days incubation at 37 °C using 10× objective. (**c**) Rough morphotype after 3 days incubation on LB agar at 37 °C. (**d**) Rough morphotype under light microscopy after 2 days incubation at 37 °C using 10× objective.

*M. abscessus* presented as two distinct morphological variants – rough and smooth. These morphotypes were isolated from logarithmically grown culture, with a higher proportion of rough colonies present in a pellicle preparation, as seen in *M. smegmatis *. The smooth morphotype was observed to be wet, smooth, and round while the rough morphotype was dry with a rough texture (Fig. 2a, c). Using light microscopy, the two *M. abscessus* morphotypes were also easily differentiated. The smooth morphotype had a defined, dark centre and translucent, spreading edges (Fig. 2b). The rough morphotype was a consistent density, much darker than the smooth morphotype, and contained structures reminiscent of cording throughout the colony (Fig. 2d). Differences between smooth and rough *M. abscessus* colonies were observed on LB, 7H10, and DTA solid media (Figs 2 and S2), suggesting that the observed morphology differences were not due to media composition.

**Fig. 3.**
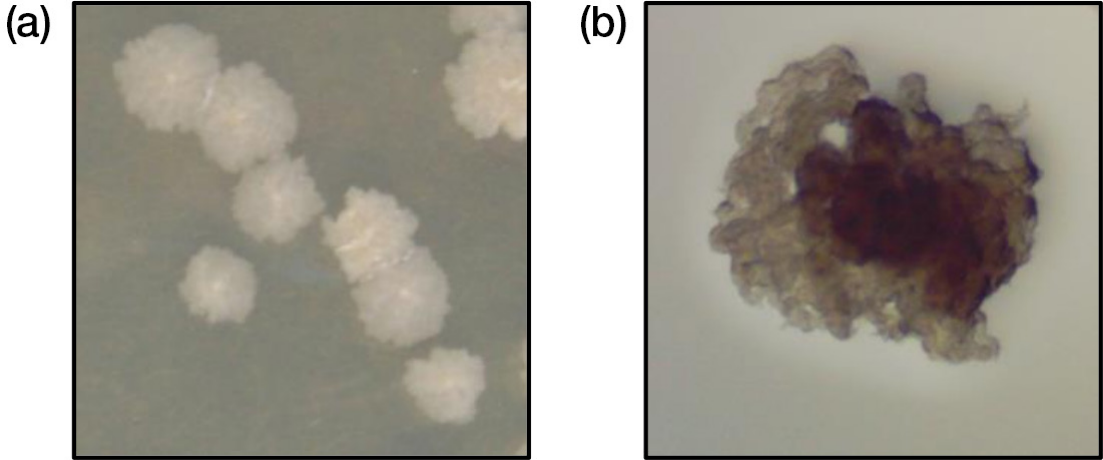
*
M. tuberculosis* was observed in only one form, similar to the rough forms of *M. smegmatis* and *M. abscessus *. (**a**) Colonies by gross observance were dry and rough, while the (**b**) colonies under light microscopy exhibited cording-like structures.

*M. tuberculosis* only formed one stable morphotype under any conditions tested, including logarithmic growth, long-term static culture, pellicle growth in both rich and minimal media, high and low pH growth, and static culture using spent media. *M. tuberculosis* formed a dense colony with structures reminiscent of cording, much like the *M. abscessus* rough morphotype (Fig. 3). This morphology was consistent on both 7H10 and DTA solid media.

### Morphotype switching

**Fig. 4.**
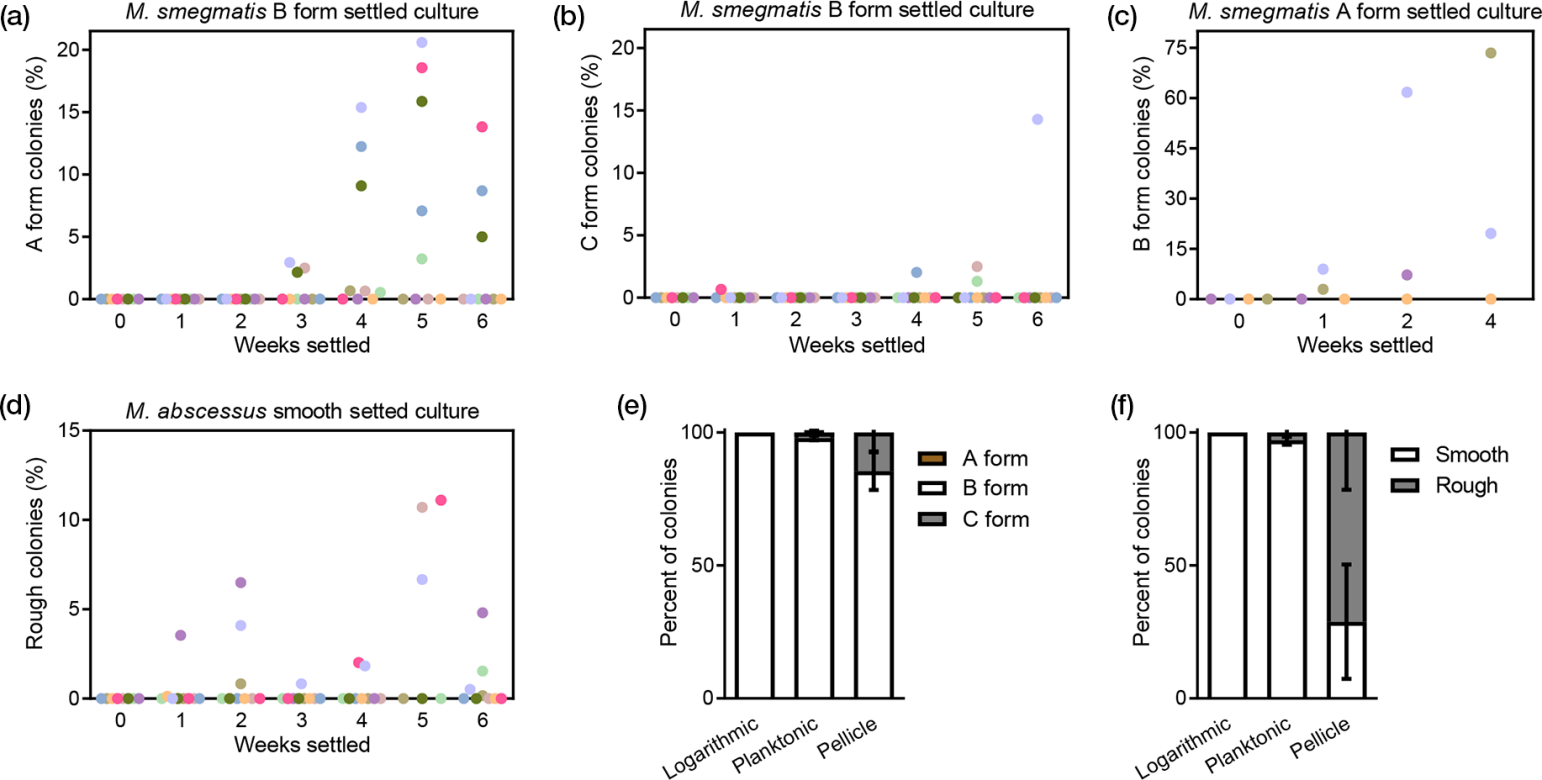
*
M. smegmatis* B morphotype switched to A (**a**) and C (**b**) morphotypes over time. The A morphotype was able to shift to the B morphotype in extended settling culture (**c**). *M. abscessus* smooth morphotype had increased number of rough colonies when settled over time (**d**). Each coloured circle represents an individual biological replicate. Both *M. smegmatis* and *M. abscessus* had increased number of rough forms in pellicle preparation after 3 weeks across four biological replicates (**e, f**). Error bars represent standard error of the mean.

All morphotypes of *M. smegmatis* and *M. abscessus* were isolated, regrown, and found to be stable; that is, they did not switch among each other upon immediate subculture (forms were sub-cultured at least five times). However, upon extended liquid culture, morphotypes were observed to switch. From extended settled culturing of the *M. smegmatis* B morphotype, both the A and C morphotypes were isolated with increasing frequency over time (Fig. 4a, b). Additionally, after extended settled culture of the A morphotype, the B morphotype was isolated with increasing frequency over time (Fig. 4c). When the B morphotype was allowed to form a pellicle, the C morphotype was generated at high frequency (Fig. 4e). The C morphotype did not switch to either the A or B morphotype under any conditions tested (Fig. S3).

As observed in *M. smegmatis,* the smooth morphotype of *M. abscessus* shifted to the rough morphotype at low frequency upon extended liquid culture (Fig. 4d). When the smooth morphotype was allowed to form a pellicle, the rough morphotype was isolated at a high frequency compared to the planktonic fraction of the same culture (Fig. 4f). Interestingly, this planktonic fraction was enriched for the rough morphotype compared to logarithmically grown culture. Extended culture of the rough morphotype was not performed in this study.

### Genome analysis of *M. smegmatis* morphotypes

Whole genome sequencing analysis was performed to determine if a genetic basis existed for the three *M. smegmatis* morphotypes. Sequencing for each morphotype was conducted on three independent isolates: one isolate from pure culture, and two isolates that had switched morphotypes (Table 1). Genomic analysis of all *M. smegmatis* forms demonstrated no reproducible sequence differences between the A and B forms. All C forms had a frameshift mutation in *MSMEG_0400* compared to the A and B forms which could be causative of a form shift. This mutation involved the addition of a guanine at position 449 888 in a string of guanines. Of note, the A and B forms were able to convert between one another, however the C form was not observed to shift back to the B form under any conditions tested.

### *M. smegmatis* A form grew slower than B and C forms

**Fig. 5.**
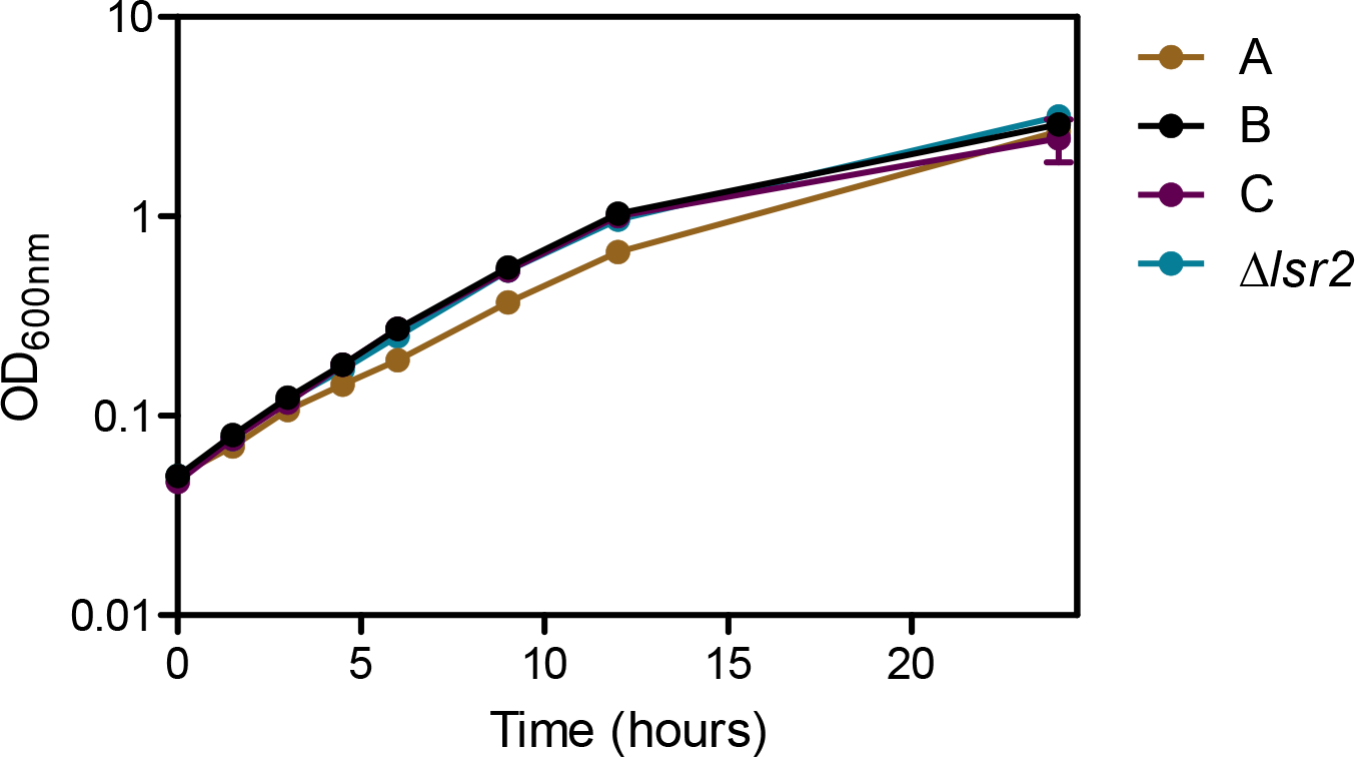
*
M. smegmatis* A morphotype grew more slowly compared to other morphotypes. OD_600nm_ was measured over 24 h of stirring aerobic growth in DTA in three biological replicates. Error bars represent standard error of the mean. Doubling times of B and C morphotypes as well as *M. smegmatis* Δ*lsr2* were the same, A morphotype doubling time was significantly longer (*P*<0.001).

To determine if the morphological changes observed highlighted more broad phenotypic alterations, growth rates were determined for each form in *M. smegmatis *. Aerated cultures of each *M. smegmatis* form were stirred for 24 h with a starting OD of 0.05. The A and B cultures remained homogenous while the C culture began to visibly clump after it reached mid-logarithmic phase growth. The B and C forms grew comparably in logarithmic phase with doubling times of 2.7 h for both. The A form grew more slowly, with a doubling time of 3.1 h (*P*<0.001). Growth slowed for all forms after 12 h, with the A form slowing the least (Fig. 5).

### Rough forms generated more robust biofilm or pellicle in both *M. smegmatis* and *M. abscessus*

**Fig. 6.**
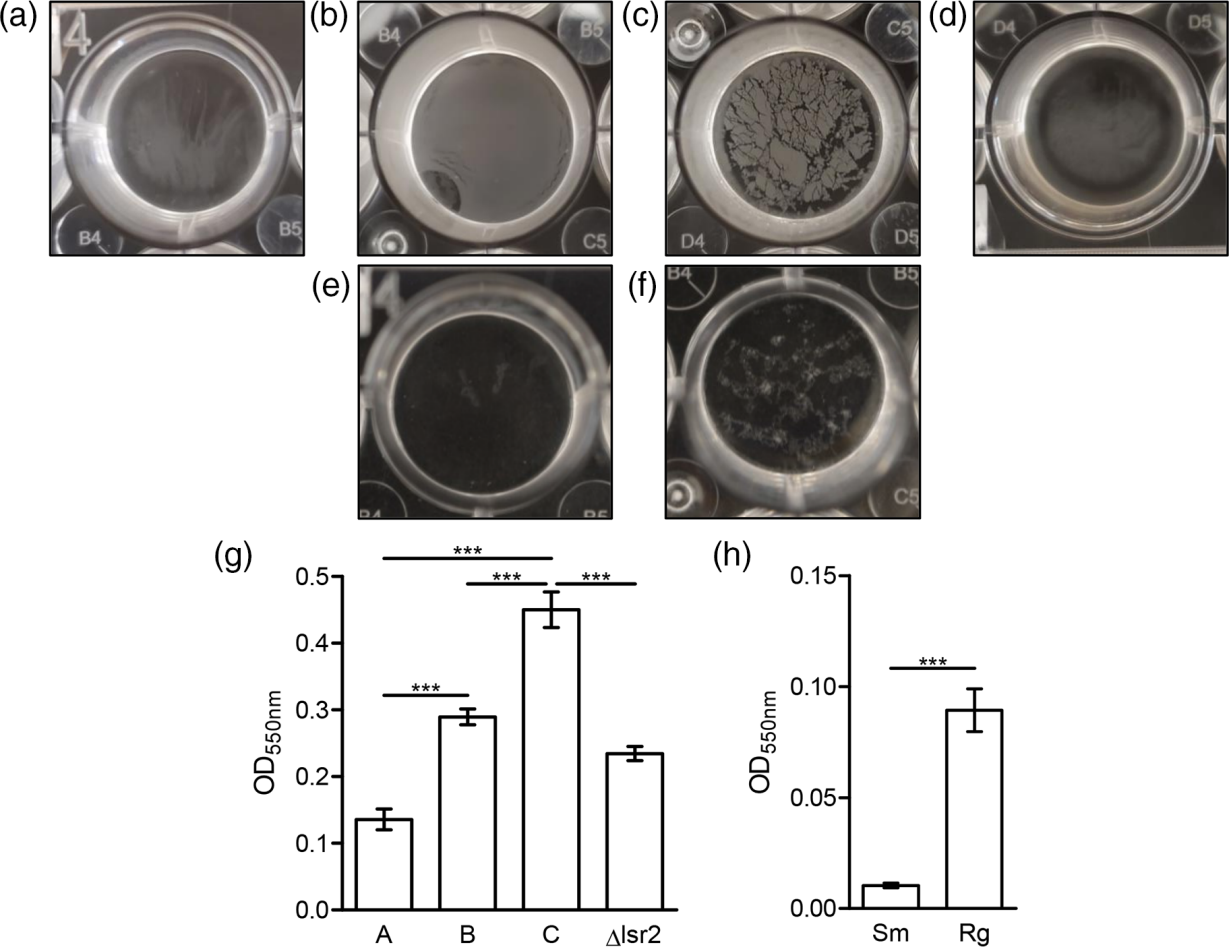
The A morphotype was deficient in biofilm formation while the C morphotype produced most robust biofilm. Dried biofilms of *M. smegmatis* A (**a**), B (**b**), C (**c**) morphotypes, and *M. smegmatis* Δ*lsr2* (**d**). *M. abscessus* dried biofilms of smooth (**e**) and rough (**f**) morphotypes. Quantification of *M. smegmatis* biofilm (**g**) and *M. abscessus* biofilm (**h**) using Gram safranin (*** *P*≤0.001). Images are representative of three biological replicates.

Biofilm formation has been noted to differ between smooth and rough forms in mycobacteria [42]. To assess the ability of our *M. smegmatis* and *M. abscessus* forms to generate biofilms, we performed a Gram safranin biofilm assay, which quantifies submerged biofilm as well as pellicle, which is the biofilm forming at the air-liquid interface. We were primarily interested in the initiation of biofilm formation, as switching to improved pellicle-forming morphotypes occurred during extended pellicle formation. While all forms were able to produce biofilms, the C form in *M. smegmatis* and the rough form in *M. abscessus* produced the most robust biofilms, likely due to pellicle formation (Fig. 6). When *M. smegmatis* biofilm formation was quantified, the A form produced the least biofilm (OD_550_ 0.14), whereas the C form produced the most biofilm (OD_550_ 0.45). The B form had intermediate biofilm generation (OD_550_ 0.29). In *M. abscessus *, the smooth form produced a slight biofilm (OD_550_ 0.02) after 2 days incubation while the rough form produced a more robust biofilm (OD_550_ 0.10) (*P*<0.001 for all comparisons). In both *M. smegmatis* and *M. abscessus *, rough forms were noted to produce a visible pellicle, which was quantified in this assay along with submerged biofilm. The smooth forms were unable to pellicle, resulting in lower safranin staining. While biofilm formation as a whole was substantially different between the two species, within-species comparisons show significant differences between the rough and smooth forms.

### *M. smegmatis* C form completely lacked sliding motility and glycopeptidolipids

**Fig. 7.**
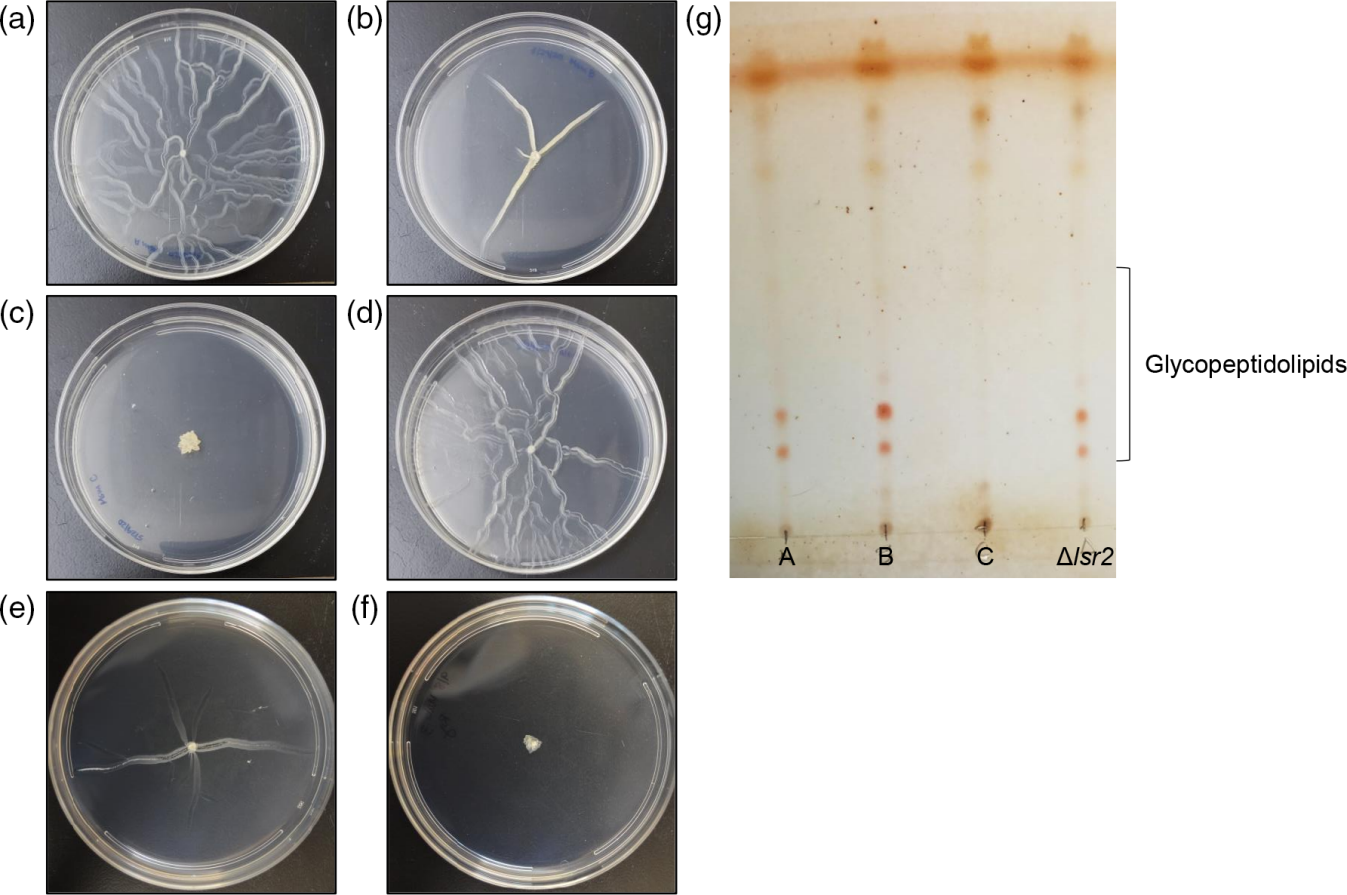
*
M. smegmatis* C form was devoid of sliding motility and glycopeptidolipid production. *M abscessus* rough form had no sliding motility. Motility agar 2 weeks after inoculation with *M. smegmatis* (**a**) A, (**b**) B, and (**c**) C morphotypes, (**d**) *M. smegmatis* Δ*lsr2*, (**e**) *M. abscessus* smooth, and (**f**) *M. abscessus* rough morphotypes. Images are representative of three biological replicates. (**g**) Thin layer chromatography showing acylated glycopeptidolipids.

Sliding motility is a unique form of motility noted in mycobacteria. Cells move away from the point of inoculation in a monolayer using pseudo-filaments along the longitudinal axes [37]. Of the three *M. smegmatis* forms, the A form showed the most pronounced sliding motility. Cells reached the edge of a standard sized petri dish, and many protrusions were observed (Fig. 7a). In the B form, protrusions originated from the point of inoculation and were almost able to reach the edge of the standard sized petri dish (Fig. 7b). The C form completely lacked sliding motility (Fig. 7c) and behaved as previously described rough mutants of *M. smegmatis* [30]. Sliding motility is thought to be dependent on glycopeptidolipid production, with the presence of glycopeptidolipids associated with greater sliding motility [37]. As hypothesized, glycopeptidolipids were absent from the C form. Interestingly, the A and B forms had similar levels of glycopeptidolipids even though there was a pronounced difference in sliding motility (Fig. 7e). In *M. abscessus *, sliding motility was observed in the smooth, but not the rough form (Fig. 7f, g) in agreement with previously published findings [32, 43].

### Morphotypes exhibited distinct susceptibilities to drug pressure

**Fig. 8.**
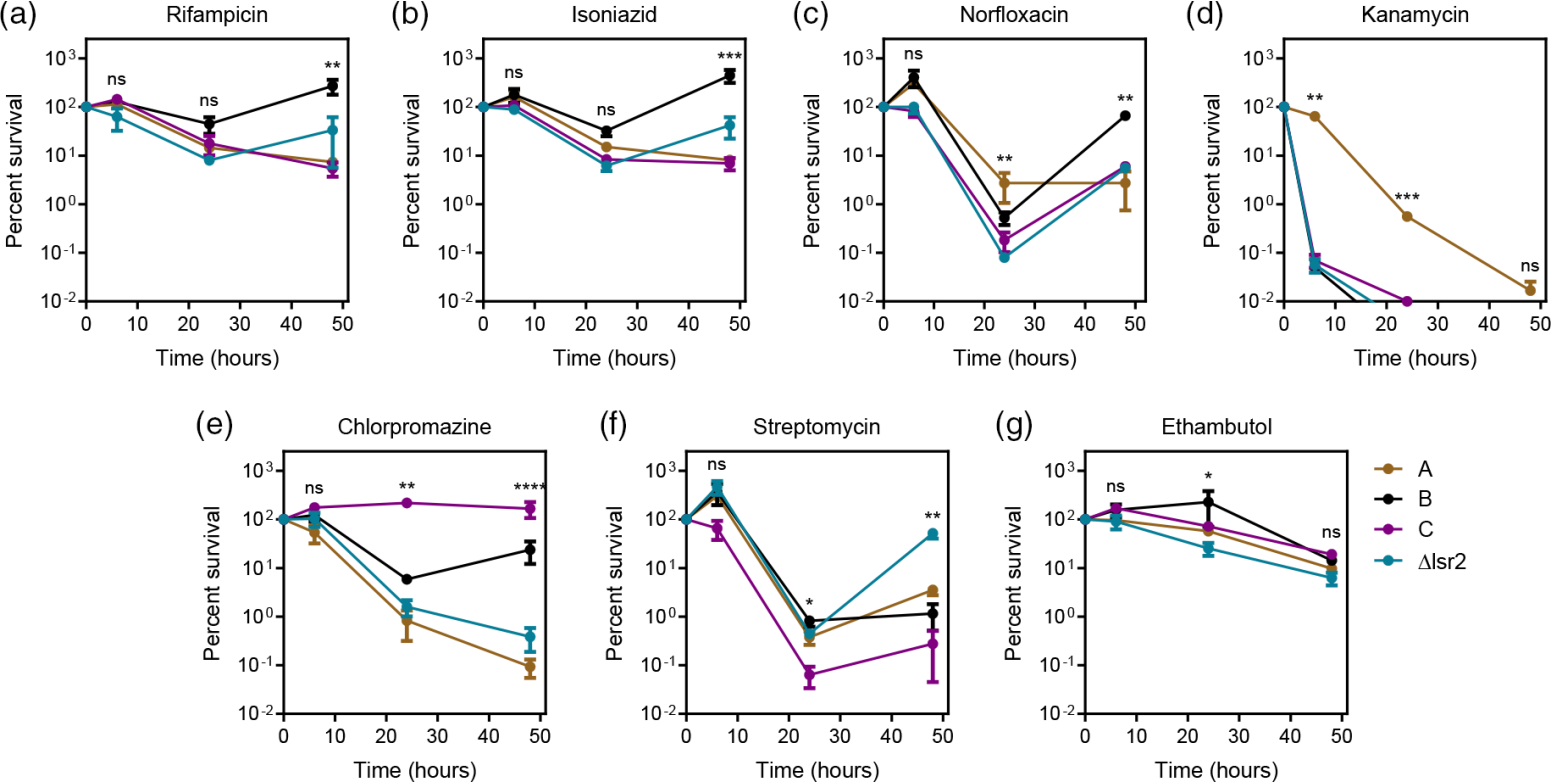
*
M. smegmatis* morphotypes responded differently to extended drug pressure. Drug concentrations were as follows: (**a**) rifampicin 12.5 µg ml^−1^, (**b**) isoniazid 125 µg ml^−1^, (**c**) norfloxacin 2.5 µg ml^−1^, (**d**) kanamycin 2.5 µg ml^−1^, (**e**) chlorpromazine 60 µg ml^−1^, (**f**) streptomycin 0.5 µg ml^−1^, and (**g**) ethambutol 2 µg ml^−1^. Gold lines represent the A morphotype, black lines represent the B morphotype, purple lines represent the C morphotype, and teal lines represent the *M. smegmatis lsr2* deletion mutant. Symbols represent the mean of three biological replicates with error bars representing standard error of the mean (* *P*≤0.05, ** *P*≤0.01, *** *P*≤0.001, **** *P*≤0.0001).

To determine if *M. smegmatis* forms respond differently to drug pressure, we determined minimum inhibitory concentration (MIC) as well as time-kill experiments with *M. smegmatis* A, B, and C forms. MICs for streptomycin, ethambutol, kanamycin, and norfloxacin were similar among all *M. smegmatis* forms, with the C form having a slightly lower MIC than other forms for isoniazid and rifampicin and a slightly higher MIC for chlorpromazine (Table 2). When the forms were exposed to drugs in time-kill experiments for 48 h (set concentration of twice the MIC for the B form), more substantial differences emerged. The B form exposed to either 12.5 µg ml^−1^ rifampicin or 125 µg ml^−1^ isoniazid died initially, but then grew by 48 h, whereas the A and C forms continued to die (Fig. 8a, b, endpoint survival *P*<0.01). When exposed to 2.5 µg ml^−1^ norfloxacin, B and C forms died and regrew by 48 h, while the A form did not regrow (Fig. 8c, endpoint survival *P*<0.01). The A form died significantly more slowly (*P*<0.001) when exposed to 2.5 µg ml^−1^ kanamycin (Fig. 8D). Chlorpromazine had perhaps the most divergent effects on the forms. When exposed to 60 µg ml^−1^ chlorpromazine, the A form decreased 110-fold in c.f.u. by 24 h and 1000-fold by 48 h. The B form decreased 18-fold in c.f.u., but by 48 h was only 4.3-fold lower than initial inoculum. The C form grew 2.2-fold by 24 h and was 1.6-fold higher than initial inoculum at 48 h (Fig. 8e, endpoint survival *P*<0.0001). The forms behaved similarly to each other when exposed to 0.5 µg ml^−1^ streptomycin or 2 µg ml^−1^ ethambutol (Fig. 8f, g). While percent survival was significantly different at some time points among forms, the trends of death and regrowth for streptomycin and slow death over time for ethambutol held true for A, B, C forms and the *lsr2* deletion mutant.

**Table 2.** Drug minimum inhibitory concentrations for *M. smegmatis* morphotypes

	Minimum inhibitory concn µg ml^−1^			
**Drug**	**A morphotype**	**B morphotype**	**C morphotype**	*** M. smegmatis* Δ*lsr2***
Isoniazid	62.5	62.5	< 62.5	62.5
Rifampicin	6.25	6.25	< 6.25	< 6.25
Streptomycin	0.25<0.5	0.25<0.5	0.25<0.5	0.25<0.5
Ethambutol	1	1	1	1
Kanamycin	1.25<2.5	1.25<2.5	1.25<2.5	1.25<2.5
Norfloxacin	1.25	1.25	1.25	0.625<1.25
Chlorpromazine	30<60	30<60	60	30<60

**Fig. 9.**
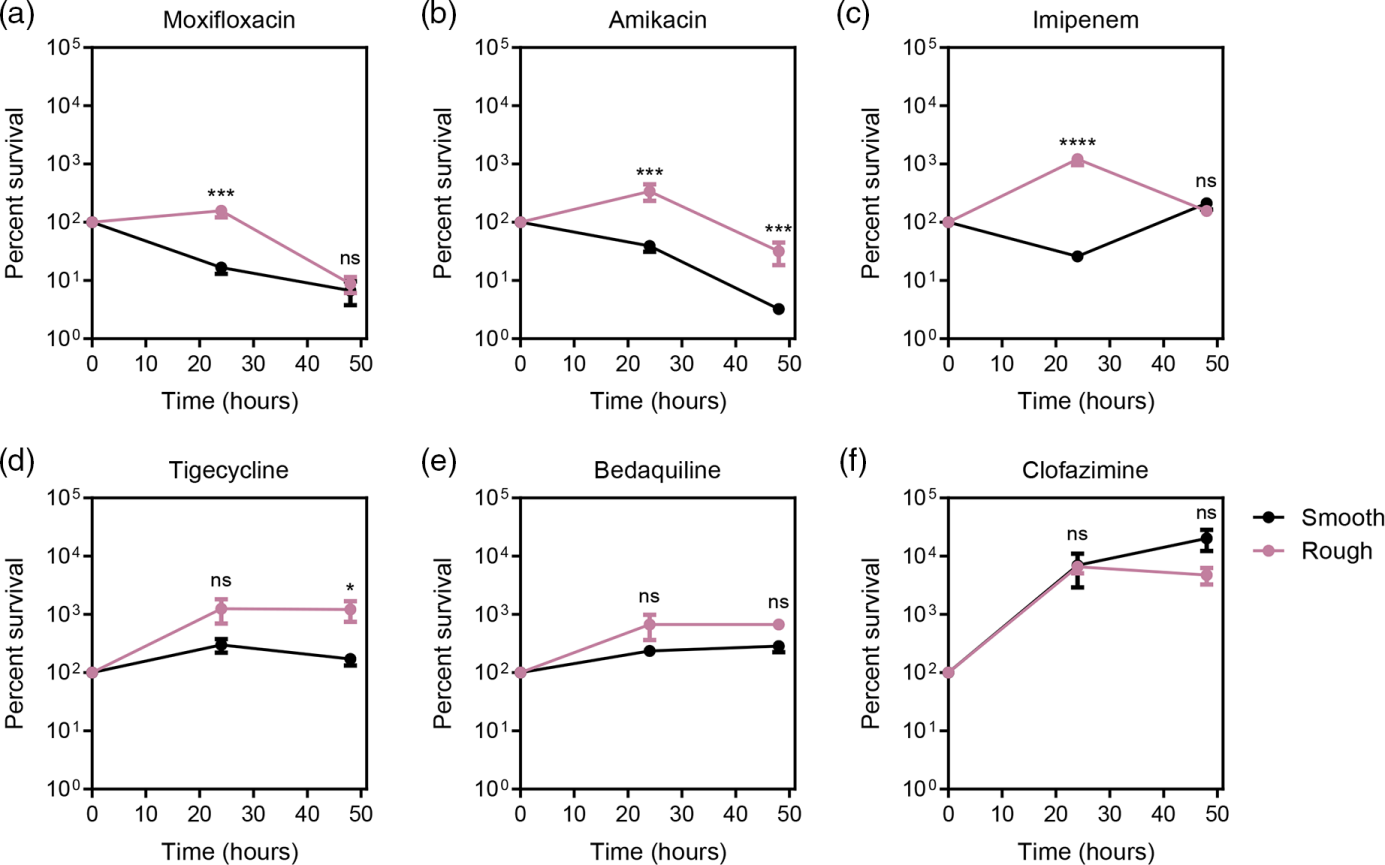
M. abscessus morphotypes responded differently to extended drug pressure. The rough form was less affected by drug than the smooth form. Drug concentrations were as follows: (A) moxifloxacin 4 µg ml^−1^, (B) amikacin 6 µg ml^−1^, (C) imipenem 2 µg ml^−1^, (D) tigecycline 4 µg ml^−1^, (E) bedaquiline 4 µg ml^−1^, and (F) clofazimine 5 µg ml^−1^. Black lines represent the smooth morphotype and pink lines represent the rough morphotype. Symbols represent the mean of three biological replicates with error bars representing standard error of the mean (* *P*≤0.05, ** *P*≤0.01, *** *P*≤0.001, **** *P*≤0.0001).

While *M. abscessus* is intrinsically tolerant or resistant to most drugs, the rough form was observed to be more tolerant to most drugs tested compared to the smooth form. When *M. abscessus* smooth and rough forms were challenged with moxifloxacin, amikacin, tigecycline, or imipenem (set concentration of twice the MIC for the smooth form), the rough form was more tolerant to the drugs. The rough form grew during the first 24 h of exposure to moxifloxacin, amikacin, imipenem, or tigecycline, while the smooth form either died or remained static (Fig. 9a-d, *P*<0.001 for moxifloxacin, amikacin, imipenem, not significant for tigecycline). Both rough and smooth forms grew when exposed to bedaquiline, however the smooth form had growth that trended slower compared to the rough form (Fig. 9e). When exposed to clofazimine, however, the smooth form appeared more tolerant than the rough form. While both forms grew in the presence of clofazimine, growth was slightly more robust in the smooth form (Fig. 9f).

### Deletion of *lsr2* shifted *M. smegmatis* population to a smooth colony morphotype

Lsr2 is a global regulatory protein in mycobacteria [35]. It has been reported that in *M. smegmatis* lacking *lsr2*, colony morphology is altered to a smooth morphotype that appeared very similar to our A morphotype [39], suggesting that Lsr2 plays a role in colony morphology. To test the effects of Lsr2 on our forms, we generated a deletion mutant of *lsr2* in an *M. smegmatis* B form background. The B form *lsr2* deletion mutant mirrored the parent strain (B form) in growth rate (Fig. 5) and biofilm formation (Fig. 6D), however the mutant had smooth colony morphology (Fig. 1m) and robust sliding motility (Fig. 7d). The *lsr2* deletion mutant and A form had similar susceptibility to chlorpromazine and streptomycin, but for all other drugs tested, the *lsr2* deletion mutant behaved more similarly to the parent B form (Fig. 8). The *lsr2* mutant decreased in c.f.u. 58-fold by 24 h and 220-fold by 48 h when exposed to chlorpromazine, much more of a robust drug response than that of the B form and more like the A form (endpoint survival *P*<0.0001). As reported previously by Kocíncová *et al.* [39], we observed that our *lsr2* mutant had high glycopeptidolipid production, as seen in both the A and B forms (Fig. 7e). These data indicate that *lsr2* may be involved in the gross colony morphology changes seen in the A morphotype; however, it appears that lack of Lsr2 is not sufficient to fully phenocopy the A form in non-morphological aspects.

## Discussion

We have demonstrated that *M. smegmatis* exists in three semi-stable morphological forms, two of which, A and B, frequently interconvert and have no detectable mutational basis. The A morphotype, which is wet, smooth, and round, had a slower rate of growth and enhanced sliding motility compared to the B and C morphotypes. It was also less likely to recover after extended drug pressure. This morphotype was primarily observed to form in static cultures that were intermittently aerated, suggesting roles for nutrient deprivation and reduced oxygen in the development of this colony morphotype. Of note, no other stable colony morphotypes were observed under any experimental conditions. Colonies with similar phenotypic characteristics have been reported when *M. smegmatis* cultures were deprived of glucose [44] and when *lsr2* was deleted [39, 45]. Lsr2 is an H-NS type nucleoid associated protein. Nucleoid associated proteins are thought to function to alter gene expression in a similar way to eukaryotic histones [46]. The complex mechanism of switching among variants may include the global regulatory protein, Lsr2, which we have demonstrated is involved in the adaptation of *M. tuberculosis* to hypoxia [35] and is involved in *M. abscessus* virulence [47]. Interestingly, while the *lsr2* deletion mutant in *M. smegmatis* did have many phenotypic similarities to the A form, the absence of Lsr2 did not completely phenocopy the A form. These data suggest that the A form has either incomplete inhibition of *lsr2* leading to a different phenotype compared to complete removal of Lsr2 or that there is another mechanism apart from Lsr2 that is responsible for the altered response to drug exposure, growth rate, and biofilm or pellicle formation. More research into the expression of Lsr2 in the A form is needed to fully understand the role of Lsr2 in morphotype heterogeneity.

The C morphotype had no sliding motility and completely lacked glycopeptidolipids, a cell wall component found in mycobacteria apart from *M. tuberculosis *. The C morphotype is more reminiscent of a typical rough form such as has been documented in multiple bacteria as a mutationally-generated form that produces more invasive disease [23, 24]. Indeed, this form did have an extra guanine in a string of guanines in *MSMEG_0400* (*mps1*), suggestive of slipped-strand mispairing, which is a mechanism of directed mutation known to generate population heterogeneity [48, 49]. *mps1* is involved in glycopeptidolipid synthesis and a mutation in this gene would likely drive a change to rough morphology [22, 27]. The C form was not observed to switch back to the B form in any conditions tested, further supporting a directed mutational mechanism of formation. Interestingly, Fujiwara *et al.* demonstrated that *M. smegmatis* J15cs, a strain with mutations in *MSMEG_0400*, productively infects macrophages, which is not possible with wild-type *M. smegmatis *. The J15cs strain completely lacks glycopeptidolipids and presents with a rough colony morphology [27], similar to our C morphotype. An important follow-up to this work would be site-directed mutagenesis to determine if the mutation seen in *MSMEG_0400* is sufficient to shift the B form to the C form. It is noteworthy that *M. tuberculosis* does not have a homolog to *MSMEG_0400* and does not produce glycopeptidolipids, further supporting the idea that the smooth phenotype in *M. smegmatis* and *M. abscessus* is due at least in part to the presence of glycopeptidolipids.

We also observed the presence of two semi-stable morphotypes in *M. abscessus* – smooth and rough. No additional stable morphotypes were observed under any experimental conditions. Our work demonstrates that the two forms could be generated predictably and reproducibly under laboratory conditions. Previous observations of the rough morphotype have been isolated from clinical samples or as a result of laboratory-generated mutation [43, 50], with reduced glycopeptidolipid production induced by nitric oxide [51]. Our experiments showed that, like the C form in *M. smegmatis *, the rough form of *M. abscessus* could be generated naturally after extended static culture of the smooth form. We also demonstrated that pellicle generation substantially increased the proportion of the rough form when compared with the planktonic fraction of the same culture. The repeatable nature of this phenomenon suggests that while the rough form may have a mutational basis, it is generated or enriched in response to specific environmental conditions which can be achieved in laboratory culture as well as in clinical settings. The generation of the *M. smegmatis* C morphotype and *M. abscessus* rough morphotype at high frequencies from pellicle preparations as well as their shared characteristics such as limited sliding motility, dry and rough colony morphology, enhanced pellicle formation, and aggregative state suggest that these forms are analogous to each other and are likely formed by a common mechanism.

This work recognizes the adaptability of *M. smegmatis* and *M. abscessus* which likely allows for survival in the diverse conditions that would be encountered by environmental organisms. Interestingly, we have found no evidence that these forms exist in *M. tuberculosis. M. tuberculosis* exists in one primary morphotype that is incapable of sliding motility or producing glycopeptidolipids, akin to the rough forms found in *M. abscessus* and *M. smegmatis *. It is tempting to speculate about the purpose of having one primary form instead of three in *M. tuberculosis.* The inability to switch to a different morphotype may reduce the environmental adaptability of *M. tuberculosis* but may confer an advantage during infection. It is interesting that as these related bacterial species shift from the strict environmental organism *M. smegmatis* to strict pathogen *M. tuberculosis *, the number of stable forms decreases. While potentially an intriguing phenomenon, research on more varied non-tuberculous mycobacteria would be required to draw reliable conclusions between number of stable forms and virulence.

In this study, we highlighted that *M. smegmatis* and *M. abscessus* can shift to different semi-stable phenotypic variants by either a non-mutational mechanism or via directed mutation. The rough morphotypes of both these bacteria formed predominantly under pellicle conditions, while the formation of smooth A morphotype of *M. smegmatis* was a rare event occurring primarily during long-term settled culture conditions. We also determined that *M. tuberculosis* does not shift between multiple stable forms. The various forms of both *M. smegmatis* and *M. abscessus* had variable drug susceptibility to multiple drug classes, suggesting that phenotypic form should be considered in drug treatment during infection or in laboratory culture. This research demonstrates that predictable form shifting occurs under standard laboratory growth conditions and not only within a host environment, indicating that specific environmental signals and a programmed response play a central role in shifting between forms.

## Supplementary Data

Supplementary material 1
