# Supplementary material for: Population heterogeneity in Mycobacterium smegmatis and Mycobacterium abscessus
Source: Microbiology (Reading). 2023 Oct 20;169(10):001402. doi: 10.1099/mic.0.001402 (PMC10634367; doi:10.1099/mic.0.001402)
Supplement: Supplementary material 1 [file mic-169-1402-s001.pdf]

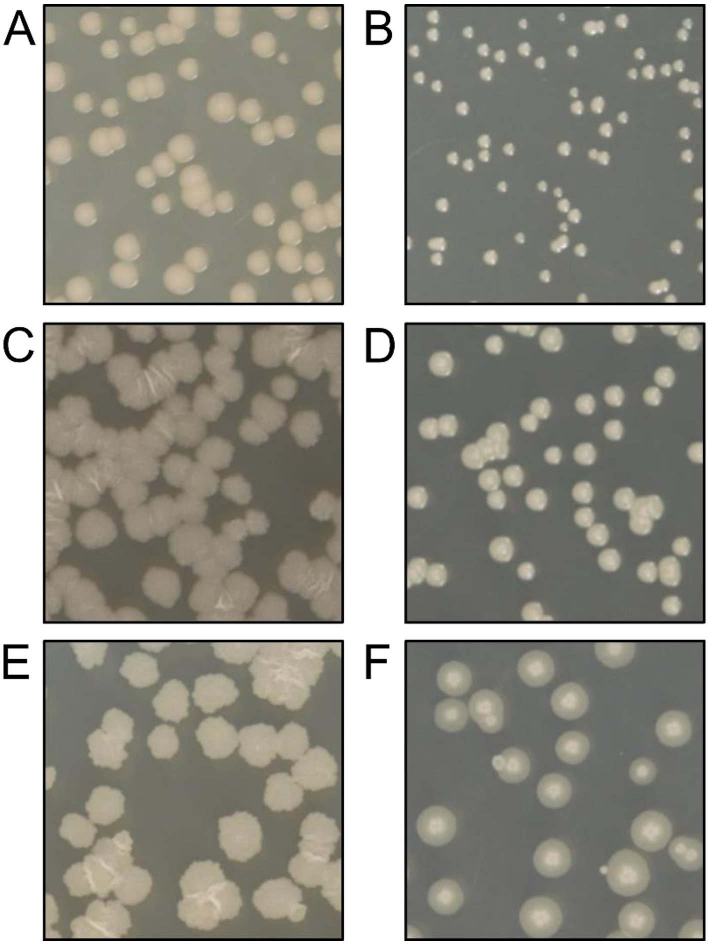

Supplemental Figure 1. *M. smegmatis* A morphotype on **(A)** 7H10 and **(B)** DTA agar; B morphotype on **(C)** 7H10 and **(D)** DTA agar; and C morphotype on **(E)** 7H10 and **(F)** DTA agar. Colonies were photographed after 3 days incubation at 37°C. Images are representative of three biological replicates.

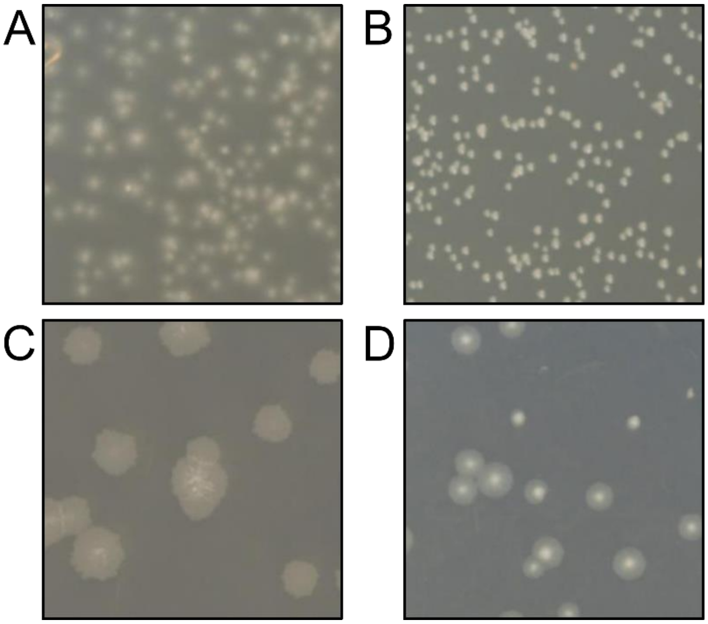

Supplemental Figure 2. *M. abscessus* smooth morphotype on **(A)** 7H10 and **(B)** DTA agar; and rough morphotype on **(C)** 7H10 and **(D)** DTA agar. Colonies were photographed after 3 days incubation at 37°C. Images are representative of three biological replicates.

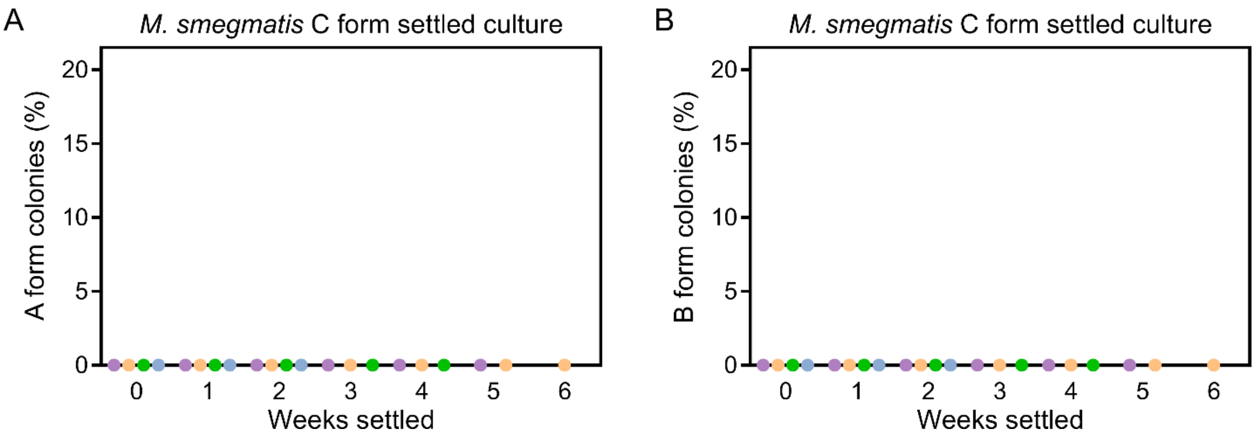

Supplemental Figure 3. *M. smegmatis* C morphotype did not switch to the **(A)** A morphotype or **(B)** B morphotype over extended static culture. Each colored circle represents an individual biological replicate.
